# Concerns about the inverse relationship between baseline HBV DNA and on-treatment hepatocellular carcinoma risk

**DOI:** 10.1172/JCI161134

**Published:** 2022-08-01

**Authors:** Shi Liu, Yongyin Li, Jian Sun

**Affiliations:** State Key Laboratory of Organ Failure Research, Guangdong Provincial Key Laboratory of Viral Hepatitis Research, Department of Infectious Diseases, Nanfang Hospital, Southern Medical University, Guangzhou, China.

**Keywords:** Hepatology, Hepatitis

## To the Editor:

We read with great interest the article by Choi et al., which concluded that baseline HBV DNA level was inversely associated with on-treatment hepatocellular carcinoma (HCC) risk in HBeAg-positive, noncirrhotic, chronic hepatitis B (CHB) patients (1). Their findings are novel, but several issues deserve discussion.

Firstly, the representativeness and potential selection bias for the study subjects are crucial, which will significantly influence the robustness and generalizability of study results. It’s uncertain whether the study subjects were enrolled consecutively, since only 2457 patients were included during the nearly ten-year enrollment at three centers. Independent cohorts with large sample size are needed to validate the conclusion.

Secondly, many confounders, like obesity, diabetes, drinking, and treatment response, were not adjusted in this study. Although the results were reproduced after excluding patients who did not achieve HBV DNA of less than 2000 IU/mL at year one, the effect of longitudinal virological response on HCC risk couldn’t be ignored. Besides, biochemical response, which has been shown to be associated with HCC risk (2), was also not adjusted.

Thirdly, it is widely accepted that high levels of HBV DNA were associated with increased HCC risk in the natural history cohort (3). However, this study showed opposite results in the nucleos(t)ide analogue–treated (NA-treated) cohort. We understand that HBeAg-positive patients in the immune-active phase, which are the target population of this study, are characterized by fluctuating but progressively decreasing HBV DNA levels, elevated alanine aminotransferase (ALT), and accumulated hepatic necroinflammation and fibrosis (4). It’s reasonable to speculate that lower baseline HBV DNA may indicate more advanced liver disease, which is a key risk factor for HCC. Although cirrhotic patients were excluded during enrollment and fibrosis 4 (FIB-4) index was adjusted in multivariate analysis, the disease severity of the enrolled patients in this study may still be heterogeneous, ranging from minimal to advanced liver damage. Without fully balancing the severity of liver damage, the inverse relationship between baseline HBV DNA level and on-treatment HCC risk might be an illusion.

Last but not least, the authors proposed that antiviral treatment can be initiated early in patients with high viral load regardless of ALT level for the prevention of HCC. However, there was no solid evidence to show the benefit of antiviral treatment in immune-tolerant patients. On the contrary, poor response to current treatments in those patients has been reported (5). Expanding current treatment indication without solid evidence would bring unnecessary potential side effects and the financial burden of long-term treatment for some patients.

## Version 1 08/01/2022

Electronic publication
